# Generalized partially functional linear model

**DOI:** 10.1038/s41598-021-02896-7

**Published:** 2021-12-06

**Authors:** Weiwei Xiao, Yixuan Wang, Haiyan Liu

**Affiliations:** 1 Department of Mathematics, North China University of Technology, Beijing, 100144 China; 2 Department of statistics, University of Leeds, Leeds, LS2 9JT UK; 3 The Alan Turing Institute, London, NW1 2DB UK

**Keywords:** Climate sciences, Health care, Mathematics and computing

## Abstract

In this paper, a generalized partially functional linear regression model is proposed and the asymptotic property of the proposed estimated coefficients in the model is established. Extensive simulation experiment results are consistent with the theoretical result. Finally, two application examples of the model are given. One is sleep quality study where we studied the effects of heart rate, percentage of sleep time on total sleep in bed, wake after sleep onset and number of wakening during the night on sleep quality in 22 healthy people. The other one is mortality rate where we studied the effects of air quality index, temperature, relative humidity, GDP per capita and the number of beds per thousand people on the mortality rate across 80 major cities in China.

## Introduction

Nowadays, more and more data, in various fields such as medicine, economics, biology and computer, are recorded in the form of curves or images. For these high-dimensional data, traditional multiple regression analysis is insufficient. Ramsay ^23^ proposed the concept of functional data. Functional data can be thought of as a real valued function defined on a compact interval. In other words, the data sampled can be represented as a function by interpolation or fitting. Indeed, functional data is different from scalar data because of its infinite dimension. Ramsay and Dalzell^24^ proposed functional data analysis. Since then, functional data analysis has been very popular among researchers. Many statistical methods such as principal component analysis, functional regression, and cluster analysis have been developed and widely used. For details on functional data analysis, see monographs Ramasy and Sliverman^25^, Ferraty and Vieu^10^, Horvath and Kokoszka^14^, Hsing and Eubank^15^, and so on.

With respect to functional regression, functional and scalar data can respectively appear in the regression model as predictors and responses. And estimation of regression coefficients is important in functional regression model. The case that response is scalar and predictors are functional is well-developed. For examples, Cardot et al.^5^ studied two estimation methods of principal component estimation and penalty spline, Cai and Hall^3^ used a principal components approach to estimate regression coefficients, Kou and Liu^19^ used a wavelet basis to estimate regression coefficients, and so on. The case where the response is scalar with functional and scalar predictors can be referred to Shin^31^ which used the least squares method to estimate regression parameters, Shin and Lee^32^ and its regression parameter estimation is based on functional principal components regression (FPCR) and the alternative is functional ridge regression (FRR) based on Tikhonov regularization, Kong et al.^18^ characterized the effects of regularization on the resulting estimators, Wang et al.^33^ used a two-step estimation method based on functional effective dimension reduction, slice inverse regression and kernel estimation to give the estimation of partial functional linear models, and gave the convergence rate of the estimation, and so on.

Generalized functional regression model has attracted more and more researchers’ interest. Müller^21^ develop generalized functional regression but only include one functional predictor and proposed a functional estimating equation which is maximizing a functional quasi-likelihood. Goldsmith et al.^11^ proposed the generalized linear mixed model and compared likelihood based and Bayesian estimation. However the response in their simulation study and application is continuous, and the corresponding asymptotic property of estimated parameters is not obtained. Crainiceanu et al.^8^ introduced generalized multilevel functional linear models. They proposed and compared two methods for inference: a two-stage frequentist approach and a joint Bayesian analysis. They studied the generalized functional linear model in which the predictive variables are both functional and scalar and the response variables are scalar. However, they do not give corresponding results for the generalized functional linear model with binary or Poisson response variables. Ieva and Paganoni^16^ proposed a generalized functional linear regression model for binary response and applied the model to the ECG signals. They studied the generalized functional linear model with binary response variables, but they did not give the corresponding asymptotic property of estimated parameters, and the predictive variables only have functional data in their application. For general response such as binary or Poisson the theory is not well-developed. In this paper, We establish generalized partially functional regression model which can deal with general response and multiple scalar and functional predictors and we develop quasi-likelihood method to estimate the regression coefficients.

Mental health problem reduces quality of life, especially during this pandemic, and sleep quality can result in severe mental health problem. Therefore study of sleep quality has received extensive attention from researchers, see Zhang^36^, Kadoya^17^ and so on. Our work is motivated by a sleep quality study aiming to investigate the relationship between sleep quality which is binary and covariates which are functional and scalar. The Multilevel Monitoring of Activity and Sleep in Healthy people (MMASH) dataset provides 24 hours of continuous beat-to-beat heart data, sleep quality, physical activity and psychological characteristics (i.e., anxiety status, stress events and emotions) for 22 healthy participants. Moreover, saliva bio-markers (i.e. cortisol and melatonin) and activity log were also provided in this dataset. In MMASH database, we have curves like heart rate, inter-beat intervals; we have scalar data like wake after sleep onset, number of awakening during the night, hormones concentrations in the saliva and so on, and we have binary response i.e. good or bad sleep quality. Our work is also motivated by another study in public health, i.e. to investigate the impact of air quality index (AQI), temperature, relative humidity (RH), GDP per capita and the number of beds per thousand people on the mortality rate across different cities in China. In particular, we are interested in studying the effect of AQI on mortality. AQI reflects the degree of air pollution and it is judged by the concentration of pollutants in the air, where the main pollutants in the air include PM2.5, PM10, carbon monoxide, nitrogen dioxide, sulfur dioxide, ozone and so on.

The paper is organized as follows. The generalized partially functional linear model is proposed in Section “Generalized partially functional linear regression”. The estimation of the regression coefficients within the generalized functional linear model is discussed in Section “[Sec Sec3]”. In Section “Asymptotic inference”, asymptotic normality of estimators is derived where the needed appropriate metrics are given. Simulation results are reported in Section “Simulation study”. Two real data examples, the sleep quality study in healthy people and the mortality rate study in major Chinese cities, are given in Section “Application”. We conclude in Section “Conclusion”. Proofs and other supplementary materials are followed in Appendix.

## Generalized partially functional linear regression

The data we observe for the *i*-th subject are $\{(X_{i 1} \left( t_{1}\right) , t_{1} \in T_{1}),\left( X_{i 2}(t_{2}\right) , t_{2} \in T_{2}), \ldots ,(X_{i d}\left( t_{d}\right) , t_{d} \in T_{d}), Z_{i}, Y_{i}\}, i=1 \ldots n$. We assume that these data are independent identically distributed (i.i.d.) copies of $(X_1, \ldots , X_d, Z, Y)$. For $j=1, \ldots , d$, the functional predictor $X_{ij}(t_{j})$ is a random curve which is observed for subject *i* and corresponds to a square integrable stochastic process on a real interval $T_{j}$, i.e. $X_{ij}(t_j)\in L^2(T_j)$. And the scalar predictor vector $Z=\left( Z_{1}, Z_{2}, \ldots Z_{q}\right)$ is a *q* dimensional random vector. The dependent variable or response $Y_{i}$ is a real-valued random variable which may be continuous or discrete (e.g. binary, count etc.).

We assume there is a known link function $g(\cdot )$ which is a monotone and twice continuously differentiable function with bounded derivatives and is thus invertible. We further assume there is a variance function $\sigma ^2(\cdot )$ which is defined on the range of the link function and is strictly positive.

We assume the following relation between $Y_{i}$ and $(X_{i1}, \ldots ,X_{id}, Z_{i})$

1 $$\begin{aligned} Y_{i}=g\left( \alpha +\sum _{j=1}^{d} \int _{T_{j}} X_{ij}\left( t_{j}\right) \beta _{j}\left( t_{j}\right) d t_{j}+Z_{i} \gamma \right) +\varepsilon _{i}, \end{aligned}$$

where $\alpha \in {\mathbb {R}}$ is the intercept, $\beta _{j}(\cdot )\in L^2(T_j)$ is the regression function corresponding to functional predictor $X_{j}$, and $\gamma =\left( \gamma _{1},\gamma _{2},\ldots ,\gamma _{q} \right) ^{T}$ is the regression coefficient vector corresponding to the scalar predictor vector *Z*. Furthermore, $\varepsilon$ is random variable from exponential family that satisfies $\mathrm {E}\left[ \varepsilon \mid X_{j}\left( t_{j}\right) , Z\right] =0$ with $\mathrm {E}\left[ \varepsilon ^{2}\right] =\mathrm {Var}\left[ \varepsilon _{i}\right] =\mathrm {Var}\left[ Y\right] =\sigma ^{2}\left( \mathrm {E}\left[ Y\right] \right)$. Therefore, the generalized partially functional linear model is determined by parameter coefficient function $\beta _{j}(\cdot )$, parameter coefficient vector $\gamma$, link function $g(\cdot )$ and variance function $\sigma ^{2}(\cdot )$.

Define linear predictors $\eta$,

2 $$\begin{aligned} \eta = \alpha + \sum _{j=1}^{d} \int _{T_{j}} X_{j}(t_{j}) \beta _{j}(t_{j}) \mathrm {d}t_{j} + Z \gamma . \end{aligned}$$

We have conditional mean $\mathrm {E}\left[ Y \mid X_{j}\left( t_{j}\right] , Z\right) = \mu =g(\eta )$ and $\mathrm {Var} \left( Y \mid X_{j} \left( t_{j} \right) , Z\right) = \sigma ^{2}(\mu )={\tilde{\sigma }}^{2}(\eta )$ for a function ${\tilde{\sigma }}^2(\eta ) = \sigma ^2(g(\eta ))$.

For simplicity, assume that both functional predictors *X*(*t*) and scalar predictors *Z* are centralized, i.e. $\mathrm {E} \left[ X_{j} \left( t_{j}\right) \right] = 0,\ j=1,2,\ldots ,d$ and $\mathrm {E}\left[ Z_{l}\right] = 0,\ l=1,2, \ldots , q$. In order to solve the problem of infinite dimension of functional data, we adopt functional principal component analysis method to reduce dimension. For functional predictor $X_{ij}(t_j)$, by Karhunen-Loeve (KL) expansion and Mercer’s theorem, it can be expanded as:

$$\begin{aligned} X_{i j}\left( t_{j}\right) =\sum _{k=1}^{\infty }\xi _{ij k} \phi _{jk}\left( t_{j}\right) , \end{aligned}$$

where $\xi _{ijk}$ is called functional principal component score and the functions $\phi _{jk}(\cdot ), k=1,2, \ldots$ are called functional principal components obtained via the covariance structure of $X_{ij}(t_j)$. Notice that $\phi _{jk}(\cdot ), k=1,2, \ldots$ forms an orthonormal basis for the function space $L^2(T_j)$ and clearly $\int _{T_j} \phi _{jk}^{2}(t_{j}) d t_{j}=1$. Then parameter coefficients $\beta _{j}(t_j)$ can be expanded as

$$\begin{aligned} \beta _{j}\left( t_{j}\right) =\sum _{k=1}^{\infty } b_{j k} \phi _{jk}\left( t_{j}\right) , \end{aligned}$$

with $$\sum b_{jk}^{2} < \infty $$.

Therefore, after plugging the above two expansions in to (1), we have:

3 $$\begin{aligned} Y_{i}=g\left( \alpha +\sum _{j=1}^{d}\sum _{k=1}^{p_j} \xi _{ijk} b_{j k}+Z_{i} \gamma \right) +\varepsilon _{i}, i=1,2, \ldots , n. \end{aligned}$$

Notice that in (3), in order to solve the difficulty caused by the infinite dimension of the functional predictors, we truncate the predictors at $p_j$, and the dimension $p_j$ increases asymptotically with $n \rightarrow \infty$.

## Estimation of $\beta$ and $\gamma$

Denote parameter vector

$$\begin{aligned} \varvec{\theta }=( b_{11},b_{12}, \ldots ,b_{1p_j},b_{21},b_{22}, \ldots ,b_{2p_j}, \ldots ,b_{d1},b_{d2}, \ldots ,b_{dp_j},\gamma _0,\gamma _1,\gamma _2, \ldots ,\gamma _q)^{T}. \end{aligned}$$

Then the maximum likelihood estimator of $\varvec{\theta }$ can be obtained by solving the score equation:

4 $$\begin{aligned} U(\varvec{\theta }) = \sum _{i=1}^{n} \frac{\left( Y_{i}-\mu _{i}\right) g^\prime \left( \eta _{i}\right) }{\sigma ^{2}\left( \mu _{i}\right) } \varvec{\omega }_{i}=0, \end{aligned}$$

where $$\eta _{i}=\alpha +\sum _{j=1}^{d} \sum _{k=1}^{p_j} b_{jk} \xi _{ijk}+Z_{i} \gamma $$, $$\mu _i=g(\eta _i),i=1,2,\ldots ,n$$ and

$$\begin{aligned} \varvec{\omega }_{i}=( \xi _{i11},\xi _{i12},\ldots ,\xi _{i1p_{1}},\xi _{i21},\xi _{i22},\ldots ,\xi _{i2p_{2}},\ldots ,\xi _{id1},\xi _{id2},\ldots ,\xi _{idp_{d}}, z_{i0},z_{i1},\ldots ,z_{iq})^{T}. \end{aligned}$$

We denote the MLE

$$\begin{aligned} \varvec{{\hat{\theta }}}=\left( {\hat{b}}_{11},{\hat{b}}_{12}, \ldots ,{\hat{b}}_{1p_j},{\hat{b}}_{21},{\hat{b}}_{22},\ldots ,{\hat{b}}_{2p_j},\ldots ,{\hat{b}}_{d1},{\hat{b}}_{d2},\ldots ,{\hat{b}}_{dp_j},{\hat{\gamma }}_0,{\hat{\gamma }}_1,{\hat{\gamma }}_2,\ldots ,{\hat{\gamma }}_q \right) ^{T} \end{aligned}$$

and therefore $\varvec{{\hat{b}}_{j}}=({\hat{b}}_{j1}, {\hat{b}}_{j2}, \ldots , {\hat{b}}_{jp_j})^{T}, \ j=1, \ldots , d$, $\varvec{{\hat{\gamma }}}=(\hat{\gamma _0},\hat{\gamma _1},\hat{\gamma _2},\ldots ,\hat{\gamma _q})^{T}$, ${\hat{\alpha }}={\hat{\gamma }}_{0}$ are the estimators of $\varvec{b_j},\ \varvec{\gamma },\ \alpha$ respectively.

We introduce the following matrices:

$$\begin{aligned} \begin{aligned} D_{0}&= D_{n, q+1}=\left( \frac{g^\prime \left( \eta _{i}\right) z_{i l}}{\sigma \left( \mu _{i}\right) }\right) _{1 \le i \le n, 0 \le l \le q},\\ D_{j}&= D_{n, p_j}=\left( \frac{g^\prime \left( \eta _{i}\right) \xi _{i j k}}{\sigma \left( \mu _{i}\right) }\right) _{1 \le i \le n, 1 \le k \le p_j}, j=1,2, \ldots , \mathrm {d},\\ D&= D_{n, q+1+ \sum _{j}^{d}p_j}={\text {diag}} \left( D_{1}, D_{2}, \ldots , D_{d},D_{0}\right) , \\ V&= {\text {diag}}\left( \sigma ^{2}\left( \mu _{1}\right) , \sigma ^{2}\left( \mu _{2}\right) , \ldots , \sigma ^{2}\left( \mu _{n}\right) \right) . \end{aligned} \end{aligned}$$

and vectors $Y=\left( Y_{1}, Y_{2}, \ldots , Y_{n}\right) ^{T}$ and $\mu =\left( \mu _{1},\mu _{2},\ldots ,\mu _{n} \right) ^{T}$. Then the score Eq. (4) can be rewritten as

$$\begin{aligned} D^{T} V^{-1 / 2}(Y-\mu )=0. \end{aligned}$$

This equation is usually solved iteratively by the method of integrated weighted least squares. Under our basic conditions in Section “Generalized partially functional linear regression”, $\frac{1}{n} \mathrm {E}(D^{T}D)$ is a fixed positive definite matrix.

## Asymptotic inference

### Generalized auto-covariance operator

Given an $L^{2}$ integrable kernel function $R(s,t):T^{2} \rightarrow R$, define the linear integral operator $A_{R}:L^{2}(\mathrm {d} s) \rightarrow L^{2}(\mathrm {d} t)$ on the Hilbert space $L^{2}(\mathrm {d}s)$ for $f \in L^{2}(\mathrm {d} t)$ by

$$\begin{aligned} \left( A_{R} f \right) (t)=\int f(s)R(s,t)\mathrm {d}s. \end{aligned}$$

An operator $A_R$ is compact self-adjoint Hilbert-Schmidt operator if

$$\begin{aligned} \int \left| R(s,t) \right| ^{2} \mathrm {d} s \mathrm {d} t < \infty , \end{aligned}$$

and can then be diagonalized.

Integral operator of special interest is the auto-covariance operator $A_{K}$ of *K* with kernel

$$\begin{aligned} K(s, t)=\mathrm {cov}(X(s), X(t))=\mathrm {E} [X(s) X(t)], \end{aligned}$$

and the generalized auto-covariance operator $A_G$ with kernel

$$\begin{aligned} G(s,t) = \mathrm {E} \left[ \frac{g^\prime (\eta )^{2}}{\sigma ^{2}(\mu )}X(s) X(t) \right] \end{aligned}$$

where $\lambda _{j,k_{1}k_{2}}$ is a non-increasing sequence of eigenvalues.

Hilbert-Schmidt operators $A_R$ generate a metric in $L^2$,

$$\begin{aligned} \begin{aligned} d_{R}^{2}(f,g)&= \int (f(t)-g(t))(A_{R}(f-g))(t) \mathrm {d}t \\&= \iint (f(s)-g(s))(f(t)-g(t))R(s,t) \mathrm {d}s \mathrm {d}t. \end{aligned} \end{aligned}$$

for $f,g \in L^2(ds)$, and given an arbitrary orthonormal basis $\{ \phi _{j},j=1,2,\ldots \}$, the Hilbert-Schmidt kernels *R* can be expressed as

$$\begin{aligned} R(s,t)=\sum _{k,l} r_{k l} \phi _{k}(s) \phi _{l}(t) \end{aligned}$$

for suitable coefficients $\{ r_{kl},k,l=1,2,\ldots \}$.

Thus, for each $X_j(t_j),\ j=1,...,d$, the correlated Hilbert-Schmidt operator of $A_G$ produces a metric in $L^2$:

$$\begin{aligned} \begin{aligned} d_{G}^{2} \left( {\hat{\beta }}_{j},\beta _{j} \right)&= \iint \left( {\hat{\beta }}_{j}(s)-\beta _{j}(s) \right) \left( {\hat{\beta }}_{j}(t)-\beta _{j}(t)\right) G(s,t) \mathrm {d}s \mathrm {d}t \\&= \iint \left( {\hat{\beta }}_{j}(s)-\beta _{j}(s)\right) \left( {\hat{\beta }}_{j}(t)-\beta _{j}(t) \right) \mathrm {E} \left[ \frac{g^\prime (\eta )^{2}}{\sigma ^{2} (\mu )}X_{j}(s)X_{j}(t)\right] \mathrm {d}s \mathrm {d}t \\&= \left( \hat{{\varvec{b}}}_{j}-{\varvec{b}}_{j} \right) ^{T} \Lambda _{j} \left( \hat{{\varvec{b}}}_{j}-{\varvec{b}}_{j} \right) , j=1,2,\ldots ,d, \end{aligned} \end{aligned}$$

where $\hat{{\varvec{b}}}_{j}=({\hat{b}}_{j 1}, {\hat{b}}_{j 2}, \ldots )^{T}$ is the estimator of ${\varvec{b}}_{j}=(b_{j1}, b_{j2}, \ldots )^{T}$, $\Lambda _{j} =(\lambda _{j,k_{1}k_{2}})_{1 \le k_{1},k_{2} \le \infty } = \left( \mathrm {E} \left[ \frac{g^\prime (\eta )^{2}}{\sigma ^{2}(\mu )}\xi _{jk_{1}} \xi _{jk_{2}}\right] \right) _{1 \le k_{1},k_{2} \le \infty }$ is a symmetric and positive definite matrix with elements to be the eigenvalues of generalized auto-covariance operator $A_G$.

For the sequence of $p_j$ truncated model (3), we have

$$\begin{aligned} d_{G}^{2}\left( {\hat{\beta }}_{j}, \beta _{j} \right) = \left( \varvec{{\hat{b}}_{j}}-\varvec{b_{j}}\right) ^{T} {\tilde{\Lambda }}_{j}\left( \varvec{{\hat{b}}_{j}}-\varvec{b_{j}}\right) +\sum _{k_1,k_2=p_j+1}^{\infty } \lambda _{j,k_{1}k_{2}} \varvec{{\bar{b}}_{j}}^{2}, j=1,2, \ldots , d, \end{aligned}$$

where $\hat{{\varvec{b}}}_{j}=({\hat{b}}_{j 1}, {\hat{b}}_{j 2}, \ldots , {\hat{b}}_{j p_j})^{T}$ is the estimator of ${\varvec{b}}_{j}=(b_{j1}, b_{j2}, \ldots , b_{jp_j})^{T}$, and here,

$$\begin{aligned} {\widetilde{\Lambda }}_{j} =(\lambda _{j,k_{1}k_{2}})_{1 \le k_{1},k_{2} \le p_j}, \\ \lambda _{j,k_{1}k_{2}}= \mathrm {E} \left[ \frac{g^\prime (\eta )^{2}}{\sigma ^{2}(\mu )}\xi _{jk_{1}} \xi _{jk_{2}}\right] , \\ {\widetilde{\Lambda }}_{j}^{-1}=(\zeta _{j,k_{1}k_{2}})_{1 \le k_{1},k_{2} \le p_j}. \end{aligned}$$

We note that ${\widetilde{\Lambda }}_{j}=\frac{1}{n} \mathrm {E}(D_{j}^{T}D_{j})$ is a symmetric and positive definite matrix and that the inverse matrix ${\widetilde{\Lambda }}_{j}^{-1}$ exists. And $\bar{{\varvec{b}}}_{j}=\left( b_{j(p_j+1)},b_{j(p_j+2)},\ldots \right) ^{T}$.

We note that higher-order oscillations associated with property $\sum _{k_1,k_2=p_j+1}^{\infty } \lambda _{j,k_{1}k_{2}} \bar{{\varvec{b}}}_{j}^{2}, j=1,2, \ldots , d$ contribute to the $L^{2}$ norm of the parameter functions $\beta _{j}(t_j)$, relative to the oscillations of processes $X_{j}(t_{j})$, i.e.,

$$\begin{aligned} \sum _{k_1,k_2=p_j+1}^\infty \lambda _{j, k_{1}k_{2}} \bar{{\varvec{b}}}_{j}^{2}=o\left( {\frac{\sqrt{p_j}}{n}} \right) , \end{aligned}$$

The specific proof follows by combining the results of corollary 4.1 of Müller (2005).

To derive the asymptotic property of $d_{G}^{2}\left( {\hat{\beta }}_{j}, \beta _{j}\right)$, in addition to the basic assumptions in Section “Generalized partially functional linear regression” and usual conditions on variance and link functions, we require some technical conditions which restrict the growth of $p_j$ and the independence of $X_{j}$ and *Z*. The basic model assumption is as follows:

1. The link function *g* is monotone, invertible and has two continuous bounded derivatives with $\Vert g^{\prime }(\cdot ) \Vert \le c$, $\Vert g^{\prime \prime }(\cdot ) \Vert \le c$ for a constant $c \ge 0$. The variance function $\sigma ^{2}(\cdot )$ has a continuous bounded derivative and there exists a $\delta >0$ such that $\sigma ^{2}(\cdot ) > \delta$.
2. The fourth moment of $X_{j}$ is finite, i.e. $\mathrm {E}\left[ \int _{T}\left\{ X_{j}(t)\right\} ^{4}\mathrm {d}t\right] <\infty$.
3. $Z=(Z_{1},\ldots , Z_{q})$ and $(X_{1}(t),X_{2}(t),\ldots ,X_{d}(t))$ are independent of each other.
4. The number of truncated terms $p_j$ in the sequence of approximating $p_j$ truncated model (3) satisfies $p_j \rightarrow \infty$ and $p_{j} n^{-\frac{1}{4}} \rightarrow 0$ as $n \rightarrow \infty$.

#### Remark 1

In the assumption (A1), because the link function *g* we defined is continuous, its first derivative is bounded. And its second derivative is bounded in order to make sure that the Hessian matrix is a meaningful existence when we prove corresponding asymptotic property. In the assumptions (A2) and (A3), the point is to simplify our proof, and we do not have to worry about the interactions between the predictors. As shown in the assumption (A4), the truncation $p_j$ goes to infinity, but in order for lemmas to hold and for the rate of convergence to be even faster, we have to $p_{j}n^{-\frac{1}{4}} \rightarrow 0$ to control the rate at which $p_j$ goes to infinity. Since *d* and *q* represent finite number of predictors, then $(q+1+\sum _{j=1}^{d}p_{j})n^{-\frac{1}{4}} \rightarrow 0$.

### Asymptotic convergence of $\beta _{j}(t)$ and $\gamma$

#### Lemma 1

As $$n \rightarrow \infty $$, assumptions (A1)–(A4)

$$\begin{aligned} \left\| \sqrt{n}(\hat{\varvec{\theta }}-\varvec{\theta })-\left( \frac{D_{j}^{T} D_{j}}{n}\right) ^{-1} \frac{U(\varvec{\theta })}{\sqrt{n}} \right\| _{2}=o(1). \end{aligned}$$

#### Remark 2

Lemma 1 essentially implies $\sqrt{n}(\hat{\varvec{\theta }}-\varvec{\theta }) \sim \left( \frac{D_{j}^{T} D_{j}}{n}\right) ^{-1} \frac{U(\varvec{\theta })}{\sqrt{n}}$.

#### Lemma 2

Under the assumptions (A1)–(A4), we have that

$$\begin{aligned} \left\| \Psi _{nj}^2-I_{nj} \right\| _2^2=O \left( \frac{1}{p_j} \right) . \end{aligned}$$

where $\Psi _{nj}$ are $p_j \times p_j$ matrices

$$\begin{aligned} \Psi _{nj}={\widetilde{\Lambda }}_{j}^{\frac{1}{2}} \left( \frac{D_j^T D_j}{n}\right) ^{-1} {\widetilde{\Lambda }}_{j}^{\frac{1}{2}} \end{aligned}$$

and $I_{nj}$ are $p_j \times p_j$ diagonal matrices.

#### Theorem 1

If the basic conditions in Section “Generalized partially functional linear regression” and assumptions (A1)–(A4) are satisfied, then

$$\begin{aligned} \left( \begin{array}{c} \frac{ nd_{G}^2 \left( {\hat{\beta }}_{1},\beta _{1}\right) -p_{1}}{\sqrt{2p_1}} \\ \frac{ nd_{G}^2 \left( {\hat{\beta }}_{2},\beta _{2}\right) -p_{2}}{\sqrt{2p_2}} \\ \vdots \\ \frac{ nd_{G}^2 \left( {\hat{\beta }}_{d},\beta _{d}\right) -p_{d}}{\sqrt{2p_d}} \\ \sqrt{n \nu _{0}}(\gamma _{0}-{\hat{\gamma }}_{0}) \\ \sqrt{n \nu _{1}}(\gamma {1}-{\hat{\gamma }}_{1}) \\ \vdots \\ \sqrt{n \nu _{q}}(\gamma _{d}-{\hat{\gamma }}_{d}) \end{array} \right) {\mathop {\longrightarrow }\limits ^{d}} N \left( 0,{I_{a}} \right) \end{aligned}$$

where $\nu _{l}=\mathrm {E}\left[ \frac{g^{\prime }(\eta _{i})^{2}}{\sigma ^{2}(\mu _{i})}z_{il}^{2} \right] ,\ l=0,1,\ldots ,q$ and $I_{a}$ is a $\left( q+1+\sum _{j=1}^{d}p_{j} \right) \times \left( q+1+\sum _{j=1}^{d}p_{j} \right)$ identity matrix.

#### Remark 3

For $j=1,2,\ldots ,d$, each $nd_{G}^2 \left( {\hat{\beta }}_{j},\beta _{j}\right)$ obeys a asymptotically normal distribution with the mean of $p_{j}$ and the variance of $2p_{j}$. For $l=0,1,\ldots ,q$, each $\sqrt{n}(\gamma _{l}-{\hat{\gamma }}_{l})$ obeys a asymptotically normal distribution with the mean of 0 and the variance of ${\mathrm {E}\left[ \frac{g^\prime (\eta _{i})^{2}}{\sigma ^{2}(\mu _{i})}z_{il}^{2} \right] }^{-1}$. Because we assume that $(X_{i1}(t_{1}),X_{i2}(t_{2}),\ldots ,X_{id}(t_{d}),z_{i0},z_{i1},\ldots ,z_{iq})$ are independent of each other, the variance term in the jointly asymptotically normal distribution is a diagonal matrix.

#### Corollary 1

Denote the eigenvectors and eigenvalues of the matrix ${\tilde{\Lambda }}_{j}$ by

$$\begin{aligned} (e_{j,1},\lambda _{j,1}), (e_{j,2},\lambda _{j,2}), \ldots , (e_{j,p_{j}},\lambda _{j,p_{j}}), \end{aligned}$$

and let

$$\begin{aligned} {\varvec{e}}_{j,k}=(e_{j,k1},e_{j,k2},\ldots ,e_{j,kp_{j}})^T, \\ \omega _{j,k}(t)=\sum _{l=1}^{p_{j}} \phi _{j,l}(t) e_{j,k}, k=1,2,\ldots ,p_{j}. \end{aligned}$$

where $\{ \phi _{j,l}(t),l=1,2,\ldots \}$ is an orthonormal basis, which is mentioned in Section “Generalized partially functional linear regression”.

Then for large *n* and $p_{j}$ an approximate $(1-\alpha )$ simultaneous confidence band is given by

$$\begin{aligned} {\hat{\beta }}_{j}(t) \pm \sqrt{c(\alpha ) \sum _{k=1}^{p_{j}} \frac{\omega _{j,k}(t)^{2}}{\lambda _{j,k}}}. \end{aligned}$$

where $c(\alpha )=[p_{j}+\sqrt{2 p_{j}} \Phi (1-\alpha )]/n$. And when $\alpha =0.05$, then $\Phi (1-\alpha )=1.96$.

## Simulation study

We consider the case with two functional predictors and three scalar predictors and the response is binary.

For the functional predictors $X_{i1}(t_{1}), \ t_{1} \in [0,1],$ and $X_{i2}(t_{2}), \ t_{2} \in [-1,1], \ i=1,...,n$, we first generate $\xi _{ijk}, \ j=1,2$ which satisfy

$$\begin{aligned} \xi _{i1k} \sim N(0,\lambda _{1k}),\ k=1,2,3,\ \text { with } \lambda _{11}=6, \lambda _{12}=4, \lambda _{13}=2 \end{aligned}$$

and

$$\begin{aligned} \xi _{i2k} \sim N(0,\lambda _{2k}),\ k=1,2,3,\ \text { with } \lambda _{21}=6, \lambda _{22}=4, \lambda _{23}=2, \lambda _{24}=\frac{1}{2}, \lambda _{25}=\frac{1}{4}. \end{aligned}$$

Defining orthonormal basis $\phi _{1k}(t_{1}),\ t_{1}\in [0,1]$ and $\phi _{2k}(t_{2}),\ t_{2}\in [-1,1],$ which satisfy

$$\begin{aligned} \phi _{1k}(t_{1})={\left\{ \begin{array}{ll} \sqrt{2} \cos \left( k \pi t_{1} \right) , k=1,2,3\\ 0, k\geqslant 4. \end{array}\right. } \\ \phi _{2k}(t_{2})={\left\{ \begin{array}{ll} \sqrt{\frac{(4k-3)}{2}} t_{2}^{2(k-1)}, k=1,2,3,4,5\\ 0, k\geqslant 6. \end{array}\right. } \end{aligned}$$

**Figure 1:**
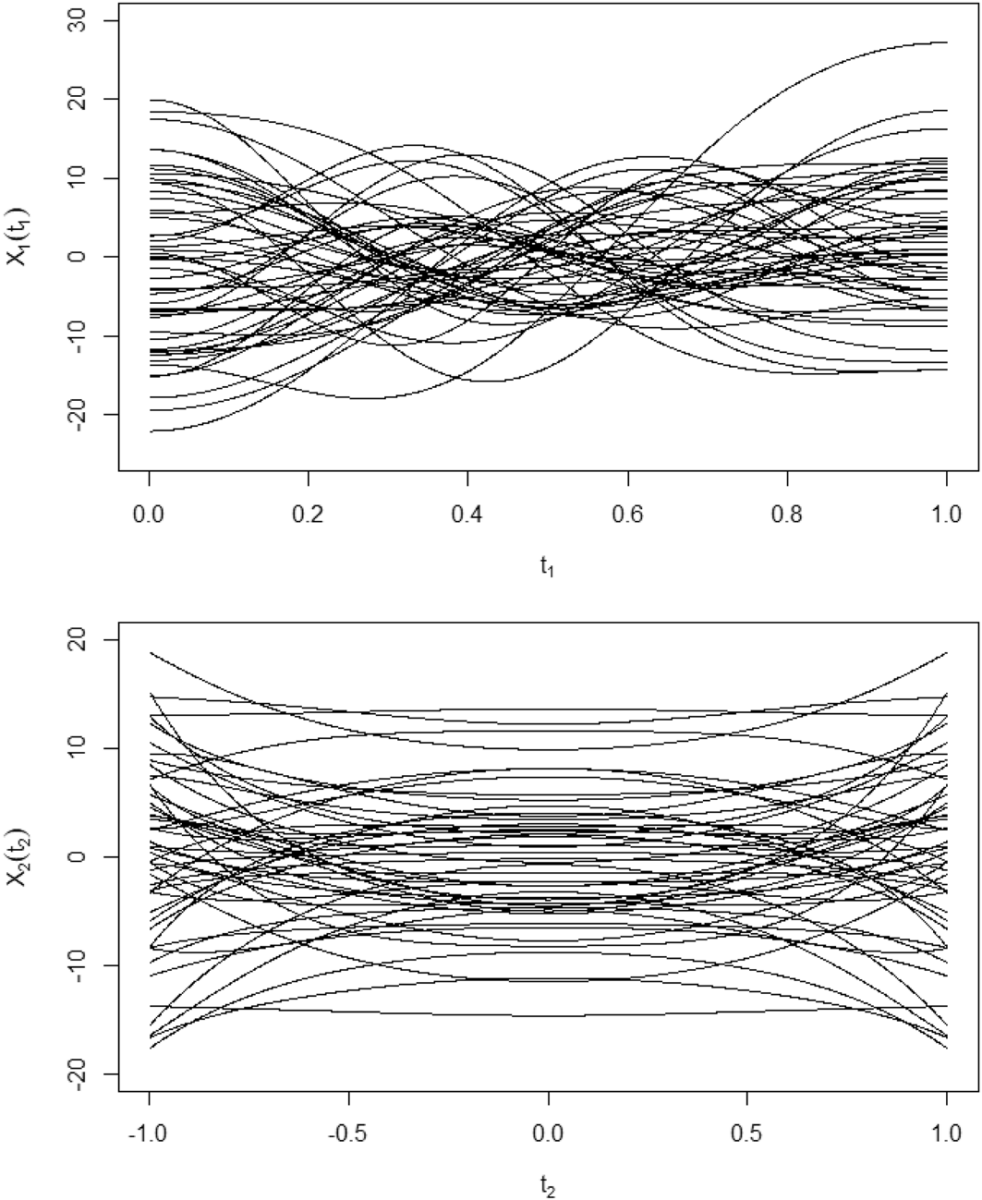
Part of the $X_{1}(t_{1})$ and $X_{2}(t_{2})$.

Therefore $X_{i1}(t_{1}), \ t_{1} \in [0,1],$ and $X_{i2}(t_{2}), \ t_{2} \in [-1,1], \ i=1,...,n$ satisfy

$$\begin{aligned} X_{i1}(t_{1})=\sum _{k=1}^{3}\xi _{i1k} \phi _{1k}\left( t_{1}\right) , \end{aligned}$$

$$\begin{aligned} X_{i2}(t_{2})=\sum _{k=1}^{5}\xi _{i2k} \phi _{2k}\left( t_{2}\right) . \end{aligned}$$

where $\phi _{jk},j=1,2$ are principal component base. Figure 1 plots part of the $X_{1}(t_{1})$ and $X_{2}(t_{2})$.

We simulate scalar predictors as follows: $z_1 \sim N(0,1)$, $z_2 \sim N(0,3)$ and $z_3 \sim B(1,0.5)$.

We assume the true regression coefficients are generated as

$$\begin{aligned} \varvec{\gamma } =(2,3,5)^T, \\ \beta _1(t_{1})=\sum _{k=1}^{3} b_{1k} \phi _{1k}(t_{1}), \\ \beta _2(t_{2})=\sum _{k=1}^{5} b_{2k} \phi _{2k}(t_{2}), \end{aligned}$$

where $$b_{1k}=2k^{2},\ b_{2k}=k^{2}$$.

Therefore the binary response is generated as follows: We define

$$\begin{aligned} p(X, Z)=g\left( \sum _{j=1}^{2} \int _{T} X_{j}\left( t_{j}\right) \beta _{j}\left( t_{j} \right) \mathrm {d} t_{j}+Z \gamma \right) , \end{aligned}$$

and choose logit link

$$\begin{aligned} g(x)=\frac{\exp (x)}{1+\exp (x)}. \end{aligned}$$

Then we generate response

$$\begin{aligned} Y(X, Z) \sim {\text {Bernoulli}}(p(X, Z), 1) \end{aligned}$$

as pseudo-Bernoulli random variable sequence with probability *p*(*X*, *Z*), when $p>0$, $Y=1$, otherwise $Y=0$. Therefore, we have a sample

$$\begin{aligned} \{(X_{i1} (t_{1}), t_{1} \in [0,1]), (X_{i2}(t_{2}), t_{2} \in [-1, 1]), Z_{i}, Y_{i}\},\ i=1 \ldots n, \end{aligned}$$

where *n* is the sample size.

In the simulation study, in order to study the asymptotic property of our estimators we choose separately $n=50,\ 500,\ 1000$, and the number of functional principal components that explain 90% of cumulative variation contribution are $p_{1}=1,\ 2,\ 2$, $p_{2}=2,\ 3,\ 4$. We do 100 simulations.

Table 1 shows the values of statistics McFadden’s pseudo $R^2$ (McFadden) and Maximum likelihood pseudo $R^2$ (r2ML) for different *n*. The simulation results show that the performance of the model gets better and better with the increase of sample size. This is consistent with our expectations.

**Table 1:** Statistics and statistical criteria under different sample sizes.

n	McFadden	r2ML
50	0.736	0.976
500	0.816	0.984
1000	0.925	0.996

**Figure 2:**
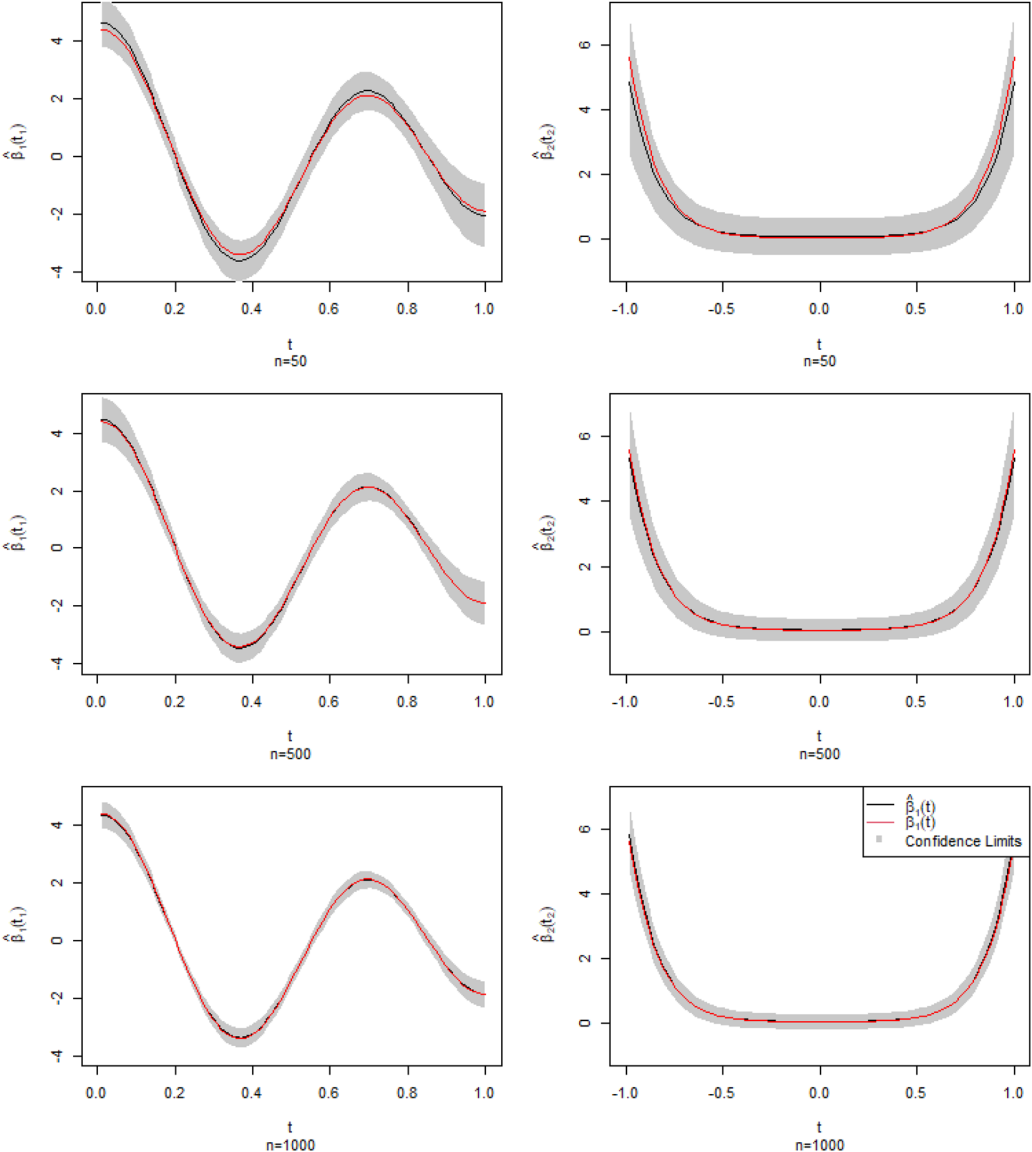
95% confidence band for the estimator ${\hat{\beta }}_{1}(t_{1})$ and ${\hat{\beta }}_{2}(t_{2})$ under different sample sizes. The red curves are theoretical $\beta _1(t_{1})$ and $\beta _{2}(t_{2})$, the black curves are the corresponding estimation and it’s the result of a simulation, the gray parts are the 95% confidence band.

Then, in order to study the performance of ${\hat{\beta }}_{1}(t_{1})$ and ${\hat{\beta }}_{2}(t_{2})$, we show the 95% confidence band under different sample sizes of the estimator ${\hat{\beta }}_{1}(t_{1})$ and ${\hat{\beta }}_{2}(t_{2})$ in Fig. 2. The red curves are true $\beta _1(t_{1})$ and $\beta _{2}(t_{2})$, the black curves are the corresponding estimations ${\hat{\beta }}_{1}(t_{1})$ and ${\hat{\beta }}_{2}(t_{2})$, the gray parts are the 95% confidence band. As we can see, as the sample size increases, the confidence band becomes narrower and narrower and the estimate (red line) gets closer to the theoretical true value (black line). Therefore, the simulation clearly indicates that the larger the sample size, the closer the estimated and true values.

In order to verify Theorem 1, we calculate the mean and variance of $nd_{G}^2 \left( {\hat{\beta }}_{j},\beta _{j}\right) ,\ j=1,2$ and $\sqrt{n}(\gamma _{l}-{\hat{\gamma }}_{l}),\ l=1,2,3$. To be specific, the theoretical values of the mean and variance of $nd_{G}^2 \left( {\hat{\beta }}_{j},\beta _{j}\right)$ are 3, 6 and 5, 10 respectively for $j=1, 2$. The theoretical values of the mean of $\sqrt{n}(\gamma _{l}-{\hat{\gamma }}_{l}),\ l=1,2,3$ are 0. Table 2 shows the sample mean and variance of $nd_{G}^2 \left( {\hat{\beta }}_{j},\beta _{j}\right) ,\ j=1, 2$ and $\sqrt{n}(\gamma _{l}-{\hat{\gamma }}_{l}),\ l=1,2,3$. From Table 2 we can see that the sample mean and sample variance of $nd_{G}^2 \left( {\hat{\beta }}_{j},\beta _{j}\right)$ tend to 3, 6 for $j=1$ and 5, 10 for $j=2$ and the sample mean of $\sqrt{n}(\gamma _{l}-{\hat{\gamma }}_{l}),\ l=1,2,3$ tend to 0 as *n* increases which verifies Theorem 1. The simulation results show that the estimation of regression coefficient functions gets better and better as the sample size increases.

**Table 2:** The mean and variance of $nd_{G}^2 \left( {\hat{\beta }}_{j},\beta _{j}\right)$ and $\sqrt{n}(\gamma _{l}-{\hat{\gamma }}_{l})$ in Theorem 1.

	*n*	Mean	Variance
$nd_{G}^2 \left( {\hat{\beta }}_{1},\beta _{1}\right)$	50	4.15	7.95
	500	3.31	6.75
	1000	3.03	6.08
$nd_{G}^2 \left( {\hat{\beta }}_{2},\beta _{2}\right)$	50	6.06	13.42
	500	5.51	11.49
	1000	5.06	10.63
$\sqrt{n}(\gamma _{1}-{\hat{\gamma }}_{1})$	50	0.19	1.26
	500	0.16	1.11
	1000	0.09	1.08
$\sqrt{n}(\gamma _{2}-{\hat{\gamma }}_{2})$	50	0.07	0.15
	500	0.04	0.13
	1000	0.03	0.11
$\sqrt{n}(\gamma _{3}-{\hat{\gamma }}_{3})$	50	0.10	5.01
	500	0.05	4.02
	1000	0.04	3.93

Table 3 shows the estimated values and corresponding standard deviations of the ${\hat{\gamma }}=(\hat{\gamma _1},\hat{\gamma _2},\hat{\gamma _3})^T$ under different sample sizes. The simulation results show that as the sample size increases, the standard deviation becomes smaller and smaller, and ${\hat{\gamma }}$ tends to the theoretical value, where the theoretical values of $\gamma =(\gamma _1,\gamma _2,\gamma _3)^T$ are 2,3,5.

**Table 3:** The estimated values and corresponding standard deviations in brackets of the estimator ${\hat{\gamma }}=(\hat{\gamma _1},\hat{\gamma _2},\hat{\gamma _3})^T$.

*n*	$\hat{\gamma _1}$	$\hat{\gamma _2}$	$\hat{\gamma _3}$
50	1.84 (0.16)	2.97 (0.06)	4.89 (0.34)
500	2.06 (0.04)	2.99 (0.02)	4.95 (0.09)
1000	2.01 (0.03)	2.99 (0.01)	5.03 (0.05)

We use GCV to demonstrate the predictive accuracy of the estimators. When $n=50,500,1000$, the corresponding GCV values are 0.104, 0.005, 0.001 which imply that as the sample size increases the prediction becomes more accurate. For details of GCV, see Roozbeh et al.^27^, Roozbeh et al.^28^, Roozbeh^29^, Amini and Roozbeh^1^ for details.

## Application

### Sleep quality

Data on activity and sleep of healthy person from PhysioNet Databases were analyzed in this section. The data were collected and provided by BioBeats (biobeats.com)^34^ in collaboration with researchers from the University of Pisa. Data were recorded on 22 healthy males in various aspects of their daily lives, such as cardiovascular responses, psychological perception, sleep quality, and exercise information.

We investigated the effects of hourly heart rate (HR), percentage of sleep time on total sleep in bed (Efficiency), wake after sleep onset (WASO) and number of awakenings during the night (Number) on sleep quality. The volunteers’ Pittsburgh Sleep Quality Questionnaire index was used as response, which follows a Bernoulli distribution where good sleep quality is represented by 1 and bad sleep quality is represented by 0. In our study, 7 men had a bad sleep quality and 15 had a good sleep quality. Efficiency, WASO and Number were used as scalar predictors. The volunteers’ hourly heart rate was used as a functional predictor. We chose $g^{-1}(x)=\frac{\exp (x)}{1+\exp (x)}$ as link function. We chose the number of functional principal components that explain 80% of cumulative variation contribution and $p=5$ .

**Figure 3:**
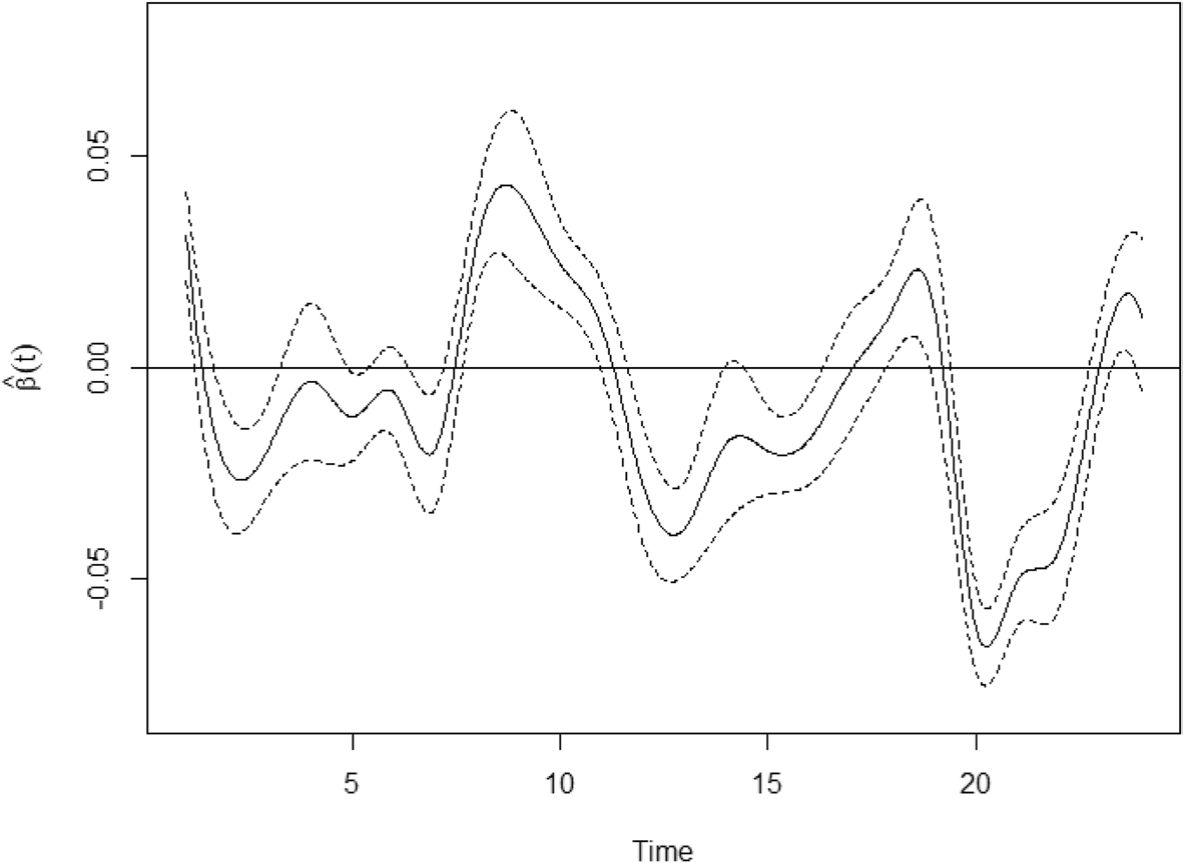
Regression coefficient function ${\hat{\beta }}(t)$ and its 95% confidence band.

By substituting the data into our model, the functional parameter coefficients ${\hat{\beta }}$ and the non-functional parameter coefficients ${\hat{\gamma }}$ can be obtained. Moreover, with respect to the prediction accuracy, the GCV is obtained: is 0.358. The results of the estimation are shown in Table 4 and Fig. 3.

**Table 4:** Parameter coefficient estimation and significance levels.

	Estimate	SE	t value	Pr( $> |t|$)	
${\hat{\gamma }}_{Efficiency}$	0.106	0.213	0.497	0.063	–
${\hat{\gamma }}_{WASO}$	0.059	0.063	0.938	0.037	–
${\hat{\gamma }}_{Number}$	− 0.143	0.007	− 0.07	0.006	**
r2ML = 0.917					

From the estimated non-functional parameter coefficient vector ${\hat{\gamma }}$ shown in Table 4, we can see that Efficiency and WASO are positively correlated with sleep quality. In other words, the greater the proportion of sleep time or the longer the first waking time after falling asleep, the better the corresponding sleep quality. On the contrary, Number is negatively correlated with sleep quality, that is, the more awakenings during the night, the poorer sleep quality.

Figure 3 shows the estimated regression coefficient function ${\hat{\beta }}(t)$ and its 95% confidence band. We can tentatively conclude that heart rate is positively correlated with sleep quality between 8:00 and 11:00. On the contrast, heart rate is mainly negatively correlated with sleep quality at other time. So, proper exercise and hard work to raise your heart rate in the morning will help improve the quality of sleep at night. While, some people may think that doing some exercise at night will improve their sleep quality because they are tired, but this is exactly the opposite. Sajjadieh (2020) used the method of time domain spectral analysis and obtained that heart rate has a negative correlation with sleep quality. But we give more insights on the dynamic and temporal behaviour of the influence of heart rate on sleep quality.

### Mortality

We have a dataset which includes daily temperature, daily relative humidity (RH), daily air quality index (AQI), GDP per capita, the number of beds per thousand people and mortality in 80 cities in China collected during 2019. A main goal of the study was to investigate the impact of temperature, RH, AQI, GDP per capita and the number of beds per thousand people on the mortality rate. We apply the proposed generalized partially functional linear model to the data for different cities.

**Figure 4:**
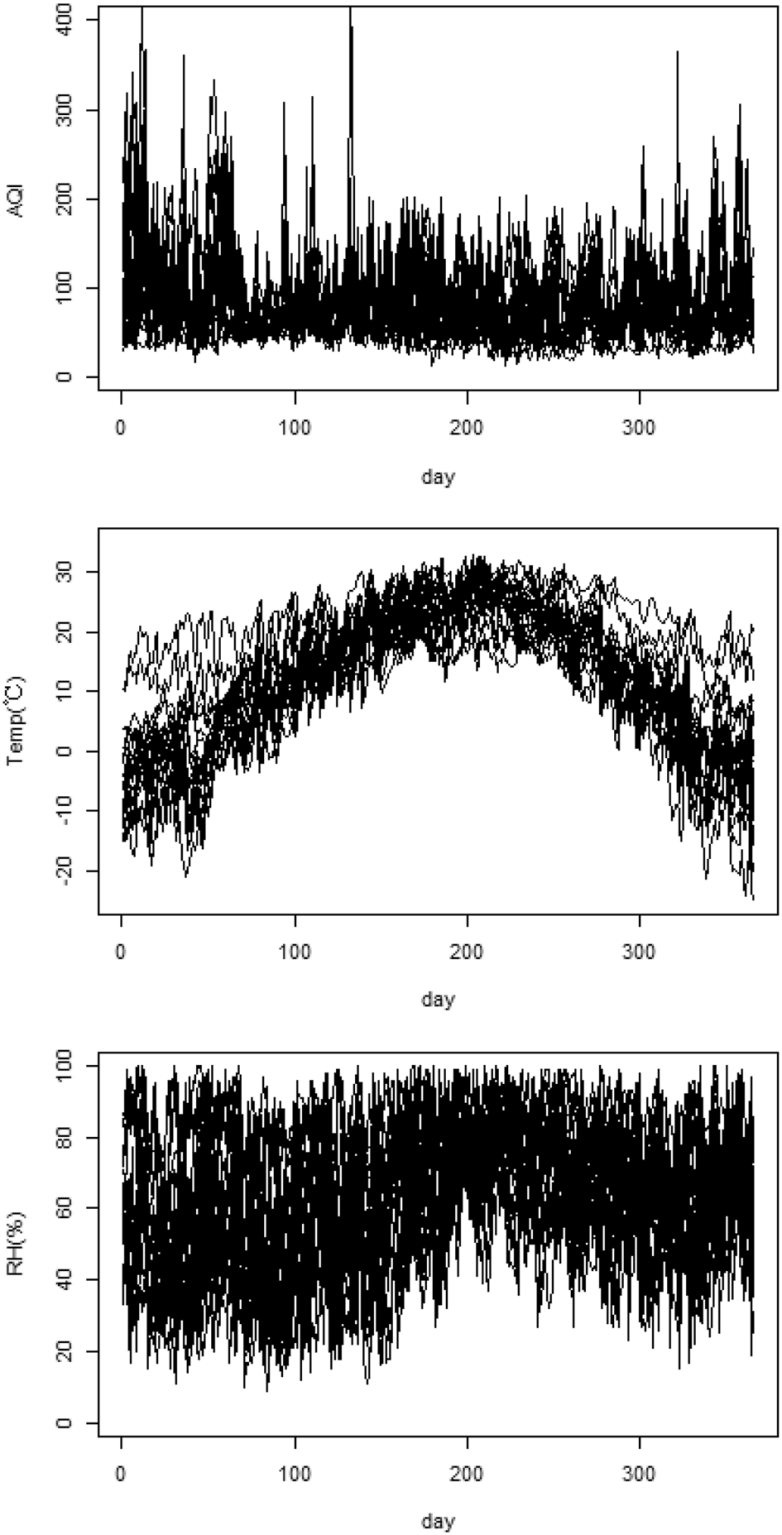
Daily AQI, temperature and RH.

We have three functional predictors: daily AQI, daily temperature and daily RH from 1 January 2019 to 31 December 2019. The two scalar predictors are GDP per capita and the number of beds per thousand people. The response is the mortality rate for each city in 2019, which is documented in the statistical bulletin of each city. Mortality follows a Bernoulli distribution where the mortality rate greater than 6‰is considered high and represented by 1, otherwise the mortality rate is low and the mortality rate is represented by 0. In our study, there are 30 cities with high mortality and 50 with low mortality. Figure 4 shows daily AQI, temperature and RH in some cities in 2019.

**Figure 5:**
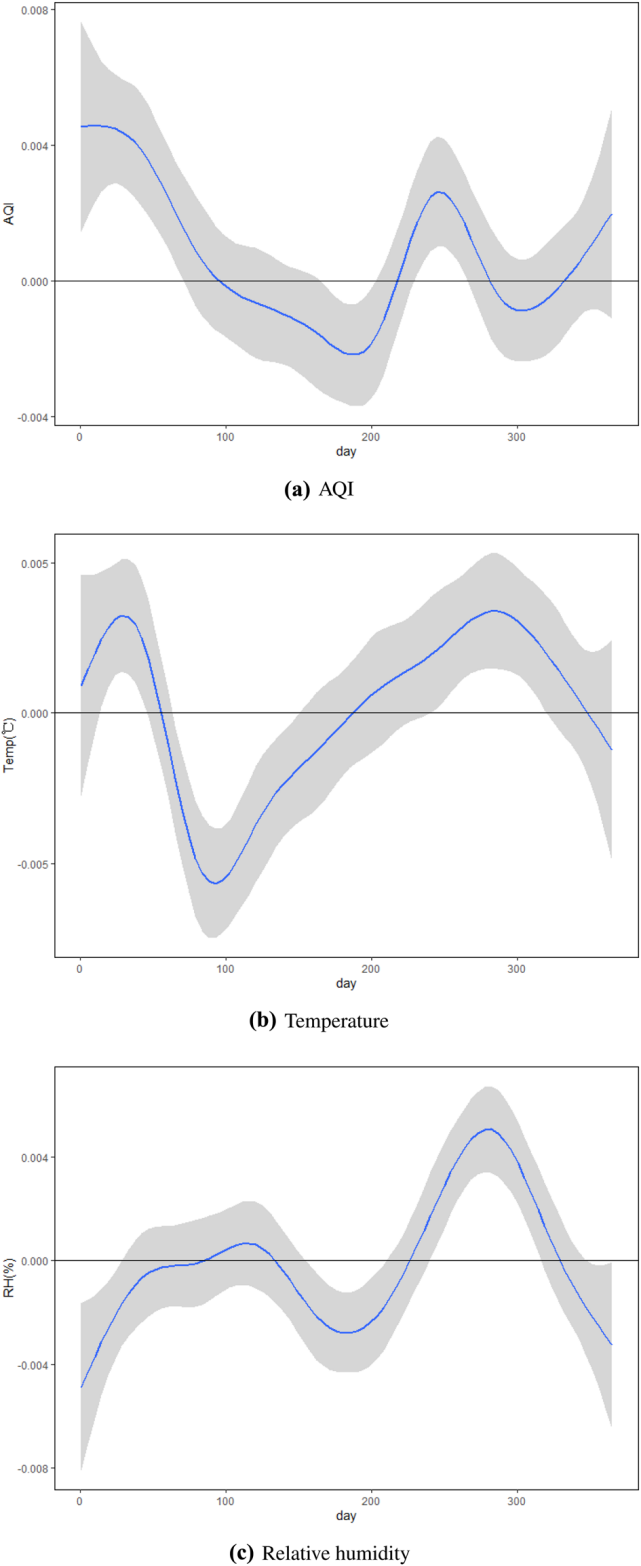
Regression coefficient function ${\hat{\beta }}(t)$ and its 95% confidence band.

We chose $g^{-1}(x)=\frac{\exp (x)}{1+\exp (x)}$ as link function. We chose the number of functional principal components that explain 75% of cumulative variation contribution, i.e. $p_{\text {AQI}}=11,\ p_{\text {Temp}}=4,\ p_{\text {RH}}=10$ as standard orthogonal basis. By substituting the data into our model, the regression coefficients of functional predictors ${\hat{\beta }}$ and regression coefficients of scalar predictors ${\hat{\gamma }}$ are shown in Table 5 and Fig. 5. Moreover, the prediction accuracy is shown by the GCV with value 0.037.

**Table 5:** Regression coefficient estimation and significance levels.

	Estimate	SE	t value	Pr( $> |t|$)	
${\hat{\gamma }}_{\text {GDP per capita}}$	− 3.181e−06	1.659e−06	− 2.165	0.035	*
${\hat{\gamma }}_{\text {Number of beds}}$	− 3.048e−01	4.718e−02	− 1.917	0.061	–

From the estimated regression coefficient vector ${\hat{\gamma }}$ shown in Table 5, we can see that GDP per capita and the number of beds per thousand people are negatively correlated with mortality rate. In other words, the greater GDP per capita or the number of beds per thousand people, the lower the mortality rate.

Figure 5 shows the estimated regression coefficient functions ${\hat{\beta }}(t)$ and their 95% confidence band. From Fig. 5, we can conclude that AQI is positively correlated with mortality in winter, early spring and fall. In other words, the higher the AQI, i.e. the worse the air conditions, the higher the death rate. Kong^18^ studied the effect of PM2.5 on mortality in US cities from 1 April 2000 to 31 August 2000. Unsurprisingly, in our study, the effect of AQI on mortality from April to August is concise with their results. For the effect of temperature on mortality, we can conclude that temperature is negatively correlated with mortality from March to May (in spring), and is positively correlated with mortality for the rest of the year. For the effect of RH on mortality, we can conclude that RH is positively correlated with mortality from August to November (in autumn), and is negatively correlated with mortality for the rest of the year. The effect of temperature and RH on mortality is very easy to use in traditional Chinese medicine (TCM) theory. In TCM, Yin and Yang are emphasized, and there is a saying that spring is born, summer is growing, autumn is converging and winter is stored. In spring, the gradually high temperature indicates that the climate is normal and Yang occurs normally. But if temperature is low in spring then Yang occurs abnormally which results in health problems. In autumn, Yang is converging, and both temperature and RH should gradually decrease. If the RH is still high, it shows that Yang cannot dive into the internal storage, floating over the outside, excessive consumption of human healthy.

## Conclusion

In this paper, we propose a generalized functional linear regression model with multiple scalar and functional predictors. We develop maximum likelihood estimators for the regression coefficients. For the functional predictors, we adopt the method of functional principal component analysis to reduce their dimensions. We then propose the generalized auto-covariance operator, based on which an appropriate measure quantifies the difference between the estimators and their true values is established. The asymptotic joint distribution of estimated regression functions is proved. For the scalar predictors, we establish a distance between the estimated value and the true value, and prove the asymptotic property of the estimated regression coefficients. The model is applied to two examples: sleep quality study and mortality rate study, and the research results clearly show that the predictors in this model explain the responses well and reveal the influence of predictors on response.

In sleep quality research, we find that Efficiency and WASO are positively correlated with sleep quality, and Number is negatively correlated with sleep quality. We also find that heart rate is positively correlated with sleep quality between 8:00 and 11:00. On the contrast, heart rate is mainly negatively correlated with sleep quality at other time. In mortality rate research, we conclude that GDP per capita and the number of beds per thousand people are negatively correlated with mortality rate. AQI is positively correlated with mortality in winter, early spring and fall. Temperature is negatively correlated with mortality from March to May (in spring). Relative humidity is positively correlated with mortality from August to November (in autumn).

The application of generalized partially functional linear model has been further extended, which lays a foundation for further research on the generalized partial-function linear model of unknown link function and variance function, predictors with interactions and variable selection with high-dimensional predictors.

## Supplementary Information

Supplementary Information.
