# Supplementary material for: Generalized partially functional linear model
Source: Sci Rep. 2021 Dec 6;11:23428. doi: 10.1038/s41598-021-02896-7 (PMC8648855; doi:10.1038/s41598-021-02896-7)
Supplement: Supplementary file 1 — Supplementary Information. [file 41598_2021_2896_MOESM1_ESM.pdf]

appendix: Proofs

The proof of Theorem 1

For the first step of the proof of Theorem 1, we adopt the usual Taylor expansion based approach for the showing asymptotic normality for an estimator which is defined through an estimating equation. Writing the Hessian of the quasi-likelihood as $J_\theta = \Delta_\theta U(\theta)$ and noting that

$$D^T D = \sum_{i=1}^{n} \frac{g'(\eta_i)^2}{\sigma^2(\mu_i)} \omega_i \omega_i^T,$$

Because we assume that $(X_{i1}(t), X_{i2}(t), \cdots, X_{id}(t), z_{i0}, z_{i1}, \cdots, z_{il})$ are independent of each other, We can easily figure out that $D^T D$ is a partitioned diagonal matrix, i.e.

$$D^T D = \begin{bmatrix} D_1^T D_1 & & & & \\ & D_2^T D_2 & & & \\ & & \ddots & & \\ & & & D_d^T D_d & \\ & & & & D_0^T D_0 \end{bmatrix}$$

For $j = 1, 2, \cdots, d$, $D_j^T D_j$ is a symmetric matrix, and $D_0^T D_0$ is a diagonal matrix.

we obtain

$$\begin{aligned} J(\theta) &= \frac{\partial U(\theta)}{\partial \theta} = \frac{\partial U(\theta)}{\partial \eta_i} \frac{\partial \eta_i}{\partial \theta} \\ &= \frac{\partial}{\partial \eta_i} \left(\sum_{i=1}^{n} \frac{(Y_i - \mu_i) g'(\eta_i)}{\sigma^2(\mu_i)} \omega_i \right) \frac{\partial \eta_i}{\partial \theta} \\ &= -\sum_{i=1}^{n} \frac{g'^2(\eta_i) \omega_i \omega_i^T}{\sigma^2(\mu_i)} + \sum_{i=1}^{n} \left(\frac{g''(\eta_i)}{\sigma^2(\mu_i)} - \frac{g'^2(\eta_i) \sigma^{2'}(\mu_i)}{\sigma^4(\mu_i)} \right) (Y_i - g(\eta_i)) \omega_i \omega_i^T \\ &= -D^T D + R. \end{aligned}$$

We aim to show that the remainder term R can eventually be neglected. By a Taylor expansion, for a $\tilde{\theta}$ between θ and $\hat{\theta}$,

$$\frac{U(\theta) - U(\hat{\theta})}{\theta - \hat{\theta}} = J_{\tilde{\theta}},$$

therefore

$$\begin{aligned} U(\theta) &= J_{\tilde{\theta}} \left(\theta - \hat{\theta} \right) \\ &= [D^T D + (J_{\tilde{\theta}} - J_\theta) + (J_\theta - D^T D)](\theta - \hat{\theta}). \end{aligned}$$

This leads to

$$\begin{aligned} \sqrt{n}(\theta - \hat{\theta}) &= \sqrt{n}[D^T D + (J_{\tilde{\theta}} - J_\theta) + (J_\theta - D^T D)]^{-1} U(\theta) \\ &= \left[\frac{D^T D}{n} + \frac{(J_{\tilde{\theta}} - J_\theta)}{n} + \frac{(J_\theta - D^T D)}{n} \right]^{-1} \frac{U(\theta)}{\sqrt{n}} \\ &= \left[I + \left(\frac{D^T D}{n} \right)^{-1} \frac{(J_{\tilde{\theta}} - J_\theta)}{n} + \left(\frac{D^T D}{n} \right)^{-1} \frac{(J_\theta - D^T D)}{n} \right]^{-1} \left(\frac{D^T D}{n} \right)^{-1} \frac{U(\theta)}{\sqrt{n}}. \end{aligned}$$

According to Lemma 1, we can obtain the asymptotically prevailing term is seen to be

$$\sqrt{n}(\theta - \hat{\theta}) \sim \left(\frac{D^T D}{n} \right)^{-1} \frac{U(\theta)}{\sqrt{n}}.$$

And then we need to prove the asymptotic convergence of $\left(\frac{D^T D}{n}\right)^{-1} \frac{U(\theta)}{\sqrt{n}}$.

$$
\begin{aligned}
\left(\frac{D^T D}{n}\right)^{-1} \frac{U(\theta)}{\sqrt{n}} &= \left(\frac{D^T D}{n}\right)^{-1} \frac{D^T V^{-\frac{1}{2}}(Y-\mu)}{\sqrt{n}} \\
&= \left(\frac{D^T D}{n}\right)^{-1} \frac{D^T V^{-\frac{1}{2}}\varepsilon}{\sqrt{n}} \\
&= \left(\frac{D^T D}{n}\right)^{-1} \frac{D^T \varepsilon'}{\sqrt{n}},
\end{aligned}
$$

where, $\varepsilon' = \frac{\varepsilon}{\sigma(\mu)}$ and ε' follows the standard normal distribution.

So that

$$\sqrt{n}(\theta-\hat{\theta}) \sim \left(\frac{D^T D}{n}\right)^{-1} \frac{D^T \varepsilon'}{\sqrt{n}}$$

Since $\beta_j(t)$ and γ have different data types, we divide $\sqrt{n}(\theta-\hat{\theta})$ into two terms, i.e.

$$\sqrt{n}(b_j-\hat{b}_j) \sim \left(\frac{D_j^T D_j}{n}\right)^{-1} \frac{D_j^T \varepsilon'}{\sqrt{n}},\ j=1,2,\cdots,d,$$

$$\sqrt{n}(\gamma-\hat{\gamma}) \sim \left(\frac{D_0^T D_0}{n}\right)^{-1} \frac{D_0^T \varepsilon'}{\sqrt{n}}.$$

So we first prove for $j=1,2,\cdots,d$

$$\sqrt{n}(b_j-\hat{b}_j) \sim \left(\frac{D_j^T D_j}{n}\right)^{-1} \frac{D_j^T \varepsilon'}{\sqrt{n}}.$$

Corresponding to

$$\mathscr{Z}_{nj} = \left(\frac{D_j^T D_j}{n}\right)^{-1} \frac{D_j^T \varepsilon'}{\sqrt{n}}.$$

Therefore, $\mathscr{Z}_{nj}$ is a p_j dimensional column vector for $j=1,2,\cdots,d$.

$$
\begin{aligned}
nd_G^2(\hat{\beta}_j,\beta_j) &= (\hat{b}_j-b_j)^T\widetilde{\Lambda}_j(\hat{b}_j-b_j) \\
&= \mathscr{Z}_{nj}^T\widetilde{\Lambda}_j\mathscr{Z}_{nj}.
\end{aligned}
$$

Of interest is then asymptotic distribution of $\mathscr{Z}_{nj}^T\widetilde{\Lambda}_j\mathscr{Z}_{nj}$. Defining p_j dimension vectors $\mathscr{X}_{nj}$ and $p_j \times p_j$ matrices Ψ_{nj} by

$$\mathscr{X}_{nj} = \frac{\widetilde{\Lambda}_j^{-\frac{1}{2}} D_j^T \varepsilon'}{\sqrt{n}},\ \Psi_{nj} = \widetilde{\Lambda}_j^{\frac{1}{2}} \left(\frac{D_j^T D_j}{n}\right)^{-1} \widetilde{\Lambda}_j^{\frac{1}{2}},$$

we obtain

$$\mathscr{X}_{nj}^T\Psi_{nj}^2\mathscr{X}_{nj} = \mathscr{Z}_{nj}^T\widetilde{\Lambda}_j\mathscr{Z}_{nj}.$$

We may decompose this into three terms

$$
\begin{aligned}
nd_G^2(\hat{\beta}_j,\beta_j) &= \mathscr{Z}_{nj}^T\widetilde{\Lambda}_j\mathscr{Z}_{nj} = \mathscr{X}_{nj}^T\Psi_{nj}^2\mathscr{X}_{nj} \\
&= \mathscr{X}_{nj}^T\mathscr{X}_{nj} + 2\mathscr{X}_{nj}^T(\Psi_{nj}^2-I_{nj})\mathscr{X}_{nj} + \mathscr{X}_{nj}^T(\Psi_{nj}^2-I_{nj})(\Psi_{nj}^2-I_{nj})\mathscr{X}_{nj}.
\end{aligned}
$$

We know from Lemma 2 that

$$\left\|\Psi_{nj}^2-I_{nj}\right\|_2^2 = O\left(\frac{1}{p_j}\right).$$

So

$$nd_G^2(\hat{\beta}_j,\beta_j) = \mathscr{X}_{nj}^T\mathscr{X}_{nj}.$$

Its matrix form is

$$
\begin{aligned}
\mathscr{X}_{nj} &= \frac{\widetilde{\Lambda}_j^{-\frac{1}{2}} D_j^T \varepsilon'}{\sqrt{n}} \\
&= \frac{1}{\sqrt{n}} \begin{bmatrix} \lambda_{j,11} & \lambda_{j,12} & \cdots & \lambda_{j,1p_j} \\ \lambda_{j,21} & \lambda_{j,22} & \cdots & \lambda_{j,2p_j} \\ \cdots & \cdots & \cdots & \cdots \\ \lambda_{j,p_j1} & \lambda_{j,p_j2} & \cdots & \lambda_{j,p_jp_j} \end{bmatrix}^{-\frac{1}{2}} \begin{bmatrix} \frac{g'(\eta_1)\xi_{1j1}}{\sigma(\mu_1)} & \frac{g'(\eta_2)\xi_{2j1}}{\sigma(\mu_2)} & \cdots & \frac{g'(\eta_n)\xi_{nj1}}{\sigma(\mu_n)} \\ \frac{g'(\eta_1)\xi_{1j2}}{\sigma(\mu_1)} & \frac{g'(\eta_2)\xi_{2j2}}{\sigma(\mu_2)} & \cdots & \frac{g'(\eta_n)\xi_{nj2}}{\sigma(\mu_n)} \\ \cdots & \cdots & \cdots & \cdots \\ \frac{g'(\eta_1)\xi_{1jp_j}}{\sigma(\mu_1)} & \frac{g'(\eta_2)\xi_{2jp_j}}{\sigma(\mu_2)} & \cdots & \frac{g'(\eta_n)\xi_{njp_j}}{\sigma(\mu_n)} \end{bmatrix} \begin{bmatrix} \varepsilon'_1 \\ \varepsilon'_2 \\ \vdots \\ \varepsilon'_n \end{bmatrix} \\
&= \frac{1}{\sqrt{n}} \begin{bmatrix} \zeta_{j,11} & \zeta_{j,12} & \cdots & \zeta_{j,1p_j} \\ \zeta_{j,21} & \zeta_{j,22} & \cdots & \zeta_{j,2p_j} \\ \cdots & \cdots & \cdots & \cdots \\ \zeta_{j,p_j1} & \zeta_{j,p_j2} & \cdots & \zeta_{j,p_jp_j} \end{bmatrix}^{\frac{1}{2}} \begin{bmatrix} \frac{g'(\eta_1)\xi_{1j1}}{\sigma(\mu_1)} & \frac{g'(\eta_2)\xi_{2j1}}{\sigma(\mu_2)} & \cdots & \frac{g'(\eta_n)\xi_{nj1}}{\sigma(\mu_n)} \\ \frac{g'(\eta_1)\xi_{1j2}}{\sigma(\mu_1)} & \frac{g'(\eta_2)\xi_{2j2}}{\sigma(\mu_2)} & \cdots & \frac{g'(\eta_n)\xi_{nj2}}{\sigma(\mu_n)} \\ \cdots & \cdots & \cdots & \cdots \\ \frac{g'(\eta_1)\xi_{1jp_j}}{\sigma(\mu_1)} & \frac{g'(\eta_2)\xi_{2jp_j}}{\sigma(\mu_2)} & \cdots & \frac{g'(\eta_n)\xi_{njp_j}}{\sigma(\mu_n)} \end{bmatrix} \begin{bmatrix} \varepsilon'_1 \\ \varepsilon'_2 \\ \vdots \\ \varepsilon'_n \end{bmatrix} \\
&= \begin{bmatrix} \frac{1}{\sqrt{n}} \sum_{k_2=1}^{p_j} \zeta_{j,1k_2}{}^{\frac{1}{2}} \sum_{i=1}^{n} \frac{g'(\eta_i)\xi_{ijk_2}}{\sigma(\mu_i)} \varepsilon'_i \\ \frac{1}{\sqrt{n}} \sum_{k_2=1}^{p_j} \zeta_{j,2k_2}{}^{\frac{1}{2}} \sum_{i=1}^{n} \frac{g'(\eta_i)\xi_{ijk_2}}{\sigma(\mu_i)} \varepsilon'_i \\ \vdots \\ \frac{1}{\sqrt{n}} \sum_{k_2=1}^{p_j} \zeta_{j,p_jk_2}{}^{\frac{1}{2}} \sum_{i=1}^{n} \frac{g'(\eta_i)\xi_{ijk_2}}{\sigma(\mu_i)} \varepsilon'_i \end{bmatrix}
\end{aligned}
$$

Then

$$
\begin{aligned}
\mathscr{X}_{nj}^T \mathscr{X}_{nj} &= \frac{1}{n} \sum_{k_1=1}^{p_j} \left(\sum_{k_2=1}^{p_j} \zeta_{j,k_1k_2}{}^{\frac{1}{2}} \sum_{i=1}^{n} \frac{g'(\eta_i)\xi_{ijk_2}}{\sigma(\mu_i)} \varepsilon'_i \right)^2 \\
&= \frac{1}{n} \sum_{k_1=1}^{p_j} \sum_{i_1,i_2=1}^{n} \sum_{k_{2'},k_{2''}=1}^{p_j} \varepsilon'_{i_1} \varepsilon'_{i_2} \frac{g'(\eta_{i_1})}{\sigma(\mu_{i_1})} \frac{g'(\eta_{i_2})}{\sigma(\mu_{i_2})} \xi_{i_1jk_{2'}} \xi_{i_2jk_{2''}} \zeta_{j,k_1k'_2}{}^{\frac{1}{2}} \zeta_{j,k_1k_{2''}}{}^{\frac{1}{2}} \\
&= \frac{1}{n} \sum_{i=1}^{n} \varepsilon_i'^2 \sum_{k_{2'},k_{2''}=1}^{p_j} \frac{g'(\eta_i)^2}{\sigma^2(\mu_i)} \xi_{ijk_{2'}} \xi_{ijk_{2''}} \sum_{k_1=1}^{p_j} \zeta_{j,k_1k_{2'}}{}^{\frac{1}{2}} \zeta_{j,k_1k_{2''}}{}^{\frac{1}{2}} \\
&\quad + \frac{1}{n} \sum_{i_1 \neq i_2=1}^{n} \varepsilon'_{i_1} \varepsilon'_{i_2} \frac{g'(\eta_{i_1})}{\sigma(\mu_{i_1})} \frac{g'(\eta_{i_2})}{\sigma(\mu_{i_2})} \sum_{k_{2'},k_{2''}=1}^{p_j} \xi_{i_1jk_{2'}} \xi_{i_2jk_{2''}} \sum_{k_1=1}^{p_j} \zeta_{j,k_1k_{2'}}{}^{\frac{1}{2}} \zeta_{j,k_1k_{2''}}{}^{\frac{1}{2}}
\end{aligned}
$$

Since ε' follows the standard normal distribution and

$$
\mathrm{E}\left[\frac{g'(\eta_i)^2}{\sigma^2(\mu_i)} \xi_{ijk_{2'}} \xi_{ijk_{2''}} \right] = \sum_{k_1=1}^{p_j} \lambda_{j,k_1k_{2'}}{}^{\frac{1}{2}} \lambda_{j,k_1k_{2''}}{}^{\frac{1}{2}} = \lambda_{j,k_{2'}k_{2''}}.
$$

For $j = 1,2,\cdots,d$, there is

$$
\begin{aligned}
\mathrm{E}\left[\mathscr{X}_{nj}^T \mathscr{X}_{nj} \right] &= p_j \\
\mathrm{Var}\left[\mathscr{X}_{nj}^T \mathscr{X}_{nj} \right] &= 2p_j.
\end{aligned}
$$

To sum up, it is proved that for $j = 1,2,\cdots,d$, $nd_G^2(\hat{\beta}_j, \beta_j)$ follows asymptotically normal distribution $N(p_j, 2p_j)$. So,

$$
\frac{nd_G^2\left(\hat{\beta}_j, \beta_j\right) - p_j}{\sqrt{2p_j}} \xrightarrow{d} N(0,1), \; j = 1,2,\cdots,d.
$$

Then we prove

$$
\sqrt{n}(\gamma - \hat{\gamma}) \sim \left(\frac{D_0^T D_0}{n} \right)^{-1} \frac{D_0^T \varepsilon'}{\sqrt{n}}.
$$

Corresponding to

$$\mathscr{Z}_0 = \left(\frac{D_0^T D_0}{n}\right)^{-1} \frac{D_0^T \varepsilon'}{\sqrt{n}}.$$

We just need to do is prove the distribution of $\mathscr{Z}_0$, and its matrix form is

$$\mathscr{Z}_0 = \frac{1}{\sqrt{n}} \begin{bmatrix} \frac{1}{n}\sum_{i=1}^n \frac{g'(\eta_i)^2}{\sigma^2(\mu_i)} z_{i0}^2 & & & \\ & \frac{1}{n}\sum_{i=1}^n \frac{g'(\eta_i)^2}{\sigma^2(\mu_i)} z_{i1}^2 & & \\ & & \ddots & \\ & & & \frac{1}{n}\sum_{i=1}^n \frac{g'(\eta_i)^2}{\sigma^2(\mu_i)} z_{iq}^2 \end{bmatrix}^{-1}$$

$$\times \begin{bmatrix} \frac{g'(\eta_1)z_{10}}{\sigma(\mu_1)} & \frac{g'(\eta_2)z_{20}}{\sigma(\mu_2)} & \cdots & \frac{g'(\eta_n)z_{n0}}{\sigma(\mu_n)} \\ \frac{g'(\eta_1)z_{11}}{\sigma(\mu_1)} & \frac{g'(\eta_2)z_{21}}{\sigma(\mu_2)} & \cdots & \frac{g'(\eta_n)z_{n1}}{\sigma(\mu_n)} \\ \cdots & \cdots & \cdots & \cdots \\ \frac{g'(\eta_1)z_{1q}}{\sigma(\mu_1)} & \frac{g'(\eta_2)z_{2q}}{\sigma(\mu_2)} & \cdots & \frac{g'(\eta_n)z_{nq}}{\sigma(\mu_n)} \end{bmatrix} \begin{bmatrix} \varepsilon_1' \\ \varepsilon_2' \\ \vdots \\ \varepsilon_n' \end{bmatrix}$$

$$= \begin{bmatrix} \sqrt{n}\left(\sum_{i=1}^n \frac{g'(\eta_i)^2}{\sigma^2(\mu_i)} z_{i0}^2\right)^{-1} \sum_{i=1}^n \frac{g'(\eta_i)z_{i0}}{\sigma(\mu_i)} \varepsilon_i' \\ \sqrt{n}\left(\sum_{i=1}^n \frac{g'(\eta_i)^2}{\sigma^2(\mu_i)} z_{i1}^2\right)^{-1} \sum_{i=1}^n \frac{g'(\eta_i)z_{i1}}{\sigma(\mu_i)} \varepsilon_i' \\ \vdots \\ \sqrt{n}\left(\sum_{i=1}^n \frac{g'(\eta_i)^2}{\sigma^2(\mu_i)} z_{iq}^2\right)^{-1} \sum_{i=1}^n \frac{g'(\eta_i)z_{iq}}{\sigma(\mu_i)} \varepsilon_i'. \end{bmatrix}.$$

So, for $l = 0, 1, \cdots, q$, we have

$$\sqrt{n}(\gamma_l - \hat{\gamma}_l) \sim \sqrt{n}\left(\sum_{i=1}^n \frac{g'(\eta_i)^2}{\sigma^2(\mu_i)} z_{il}^2\right)^{-1} \sum_{i=1}^n \frac{g'(\eta_i)z_{il}}{\sigma(\mu_i)} \varepsilon_i'.$$

Because ε' follows the standard normal distribution, We can easily figure out

$$\mathrm{E}\left[\sqrt{n}(\gamma_l - \hat{\gamma}_l)\right] = 0,$$

$$\mathrm{Var}\left[\sqrt{n}(\gamma_l - \hat{\gamma}_l)\right] = \mathrm{E}\left[\sum_{i=1}^n \frac{g'(\eta_i)^2}{\sigma^2(\mu_i)} z_{il}^2\right]^{-1} = \nu_l^{-1}.$$

To sum up, it is proved that for $l = 1, 2, \cdots, q$, $\sqrt{n}(\gamma_l - \hat{\gamma}_l)$ follows asymptotically normal distribution $N\left(0, \nu_l^{-1}\right)$. So,

$$\sqrt{n\nu_l}(\gamma_l - \hat{\gamma}_l) \xrightarrow{d} N(0,1),\ l = 0, 1, \cdots, q.$$

In summary, Theorem 1 holds.

The proof of Lemma 1

To prove $\left\|\sqrt{n}(\theta - \hat{\theta}) - \left(\frac{D^T D}{n}\right)^{-1} \frac{U(\theta)}{\sqrt{n}}\right\|_2 = o(1)$, we just need to prove $\left\|\left(\frac{D^T D}{n}\right)^{-1} \frac{(J_{\tilde{\theta}} - J_\theta)}{n}\right\|_2 = o(1)$ and $\left\|\left(\frac{D^T D}{n}\right)^{-1} \frac{(J_\theta - D^T D)}{n}\right\|_2 = o(1)$.

From $J(\theta) = -D^T D + R$, we can obtain

$$E\left[\left\|\left(\frac{(J_\theta - D^T D)}{n}\right)\right\|_2^2\right] = O\left(\frac{(q + 1 + \sum_{j=1}^d p_j)^2}{n}\right) = o\left(\frac{1}{\sqrt{n}}\right) \to 0.$$

Since $\left\|g^{(v)}(\cdot)\right\| \le c < \infty, v = 1, 2,$ $\tilde{\sigma}'^2(\cdot) \le \tilde{c} < \infty$ and $0 < \delta \le \tilde{\sigma}^2(\cdot)$ according to (A1). Together with $p_j n^{-1/4} \to 0$ (A4), this implies

$$\left\|\left(\frac{D^T D}{n}\right)^{-1} \frac{(J_{\tilde{\theta}} - J_\theta)}{n}\right\|_2 = o(1).$$

Similarly

$$\left\|\left(\frac{D^T D}{n}\right)^{-1} \frac{(J_\theta - D^T D)}{n}\right\|_2 = o(1).$$

The proof of Lemma 2

The proof of lemma 2, Müller (2005) has been given, and we will directly quote its conclusion.

The proof of Corollary

According to Theorem 1, there is

$$nd_G^2(\hat{\beta}_j, \beta_j) \sim N(p_j, 2p_j),$$

and an asymptotic $(1-\alpha)$ confidence region for β_j is given by

$$\frac{nd_G^2(\hat{\beta}_j, \beta_j) - p_j}{\sqrt{2p_j}} \leq \Phi(1-\alpha).$$

So that

$$d_G^2(\hat{\beta}_j, \beta_j) = \left(\hat{b}_j - b_j\right)^T \tilde{\Lambda}_j \left(\hat{b}_j - b_j\right) \leq [p_j + \sqrt{2p_j}\Phi(1-\alpha)]/n.$$

The asympotic $(1-\alpha)$ confidence ellipsoid for $b_j \in R^{p_j}$ is

$$(\hat{b}_j - b_j)^T \left(\frac{\tilde{\Lambda}_j}{c(\alpha)}\right)(\hat{b}_j - b_j) \leq 1.$$

For simpler calculation, the confidence ellipsoid is normalized as a confidence sphere. Expressing the vectors $\hat{b}_j$, b_j in terms of the eigenvectors $e_{j,k}$ leads to the coefficients

$$\hat{b}^*_{j,k} = \sum_{l=1}^{p_j} e_{j,kl}\hat{b}_{j,l},$$

$$b^*_{j,k} = \sum_{l=1}^{p_j} e_{j,kl} b_{j,l},$$

and with

$$\omega^*_{j,k}(t) = \omega_{j,k}(t)\sqrt{\frac{c(\alpha)}{\lambda_{j,k}}}$$

the confidence ellipsoid corresponds to the sphere

$$\sum_{k=1}^{p_j}\left(\frac{(\hat{b}^*_j - b^*_j)}{\sqrt{\frac{c(\alpha)}{\lambda_{j,k}}}}\right)^2 \leq 1.$$

To obtain the confidence band, we need to maximize

$$|\sum_{k=1}^{p_j}(\hat{b}^*_j - b^*_j)\omega_{j,k}(t)| = |\sum_{k=1}^{p_j}\frac{(\hat{b}^*_j - b^*_j)}{\sqrt{\frac{c(\alpha)}{\lambda_{j,k}}}}\omega^*_{j,k}(t)|.$$

By Cauchy-Schwarz,

$$|\sum_{k=1}^{p_j}\frac{(\hat{b}^*_j - b^*_j)}{\sqrt{\frac{c(\alpha)}{\lambda_{j,k}}}}\omega^*_{j,k}(t)| \leq [\sum_{k=1}^{p_j}\omega^*_{j,k}(t)]^{1/2}$$

and the maximizing $\frac{(\hat{b}^*_j - b^*_j)}{\sqrt{\frac{c(\alpha)}{\lambda_{j,k}}}}$ must be linear dependent with the vector $\omega^*_{j,1}(t), \cdots, \omega^*_{j,p_j}(t)$, so that the Cauchy-Schwarz inequality becomes an equality. The result then follows from the definition of the $\omega^*_{j,k}(t)$.
